# Effects of modified external ventricular drainage vs. an Ommaya reservoir in the management of hydrocephalus with intracranial infection in pediatric patients

**DOI:** 10.3389/fneur.2023.1303631

**Published:** 2024-01-11

**Authors:** Liuyin Chen, Mingzhe He, Lei Shi, Yanke Yue, Pengyuan Luo, Jiangshun Fang, Na Wang, Zhenghai Cheng, Yi Qu, Zhiguo Yang, Yaning Sun

**Affiliations:** Department of Neurosurgery, Hebei Provincial Children's Hospital, Shijiazhuang, Hebei, China

**Keywords:** hydrocephalus, intracranial infection, modified external ventricular drainage, Ommaya reservoir, pediatric

## Abstract

**Background:**

Hydrocephalus with intracranial infection (HII) may cause pathological changes in brain tissue structure and irreversible damage to the nervous system. However, intracranial infection is a contraindication to ventriculo-peritoneal (VP) shunt surgery, and the prognosis is improved by early infection control and intracranial pressure reduction. This study evaluated the safety and efficacy of the Ommaya reservoir vs. modified external ventricular drainage (M-EVD) in the management of HII in pediatric patients.

**Methods:**

This retrospective controlled study included 45 pediatric patients with HII treated with an Ommaya reservoir (*n* = 24) or M-EVD (*n* = 21) between January 2018 and December 2022. Clinical outcomes, cerebrospinal fluid (CSF) test results, complications, and outcomes were compared between the Ommaya reservoir and M-EVD groups.

**Results:**

No patient died during the follow-up period. The two groups were similar regarding age, sex, admission temperature, weight, preoperative serum protein and albumin concentrations, CSF analysis (white blood cell count, glucose concentration, and protein content), and clinical symptoms (*P* > 0.05). Both groups had significant changes in the CSF test results postoperatively compared with preoperatively (*P* < 0.05). In the M-EVD group, the median days for 13 children to remove the external drainage tube and receive VP shunt was 19 days. The longest drainage tube retention time was 61 days, and there was no intracranial infection or serious complication related to the drainage tube. After the placement of the Ommaya, the median time required for CSF to return to normal was 21 days, and a total of 15 patients underwent VP shunt surgery.

**Conclusion:**

The Ommaya reservoir and M-EVD are safe and effective for pediatric patients with HII. Both methods reduce the intracranial pressure and alleviate the symptoms of hydrocephalus, although there are differences between the two methods.

## Introduction

Hydrocephalus is a common disease in young children (1). A large amount of cerebrospinal fluid (CSF) causes the ventricle to expand and results in a significant increase in ventricular pressure, which gradually aggravates the compression of brain tissue. This results in brain dysfunction, with high incidences of disability and mortality (2, 3). Surgery is an effective method to treat hydrocephalus. At present, the most common surgical method is ventriculo-peritoneal (VP) shunt surgery (4, 5). However, intracranial infection is a contraindication to VP shunt surgery because this may lead to shunt blockage and operation failure (6, 7). Furthermore, non-specific intracranial infection in pediatric patients often leads to the obstruction of CSF absorption and circulation due to exudate accumulation and meningeal adhesion, inducing acute communicating hydrocephalus. Therefore, hydrocephalus and intracranial infection are separate risk factors for brain damage (8).

Lumbar cistern drainage, conventional external ventricular drainage (C-EVD), and placement of an Ommaya reservoir reduce intracranial pressure by releasing CSF to save the patient's life in an emergency situation. However, since lumbar cistern drainage has many complications, C-EVD and Ommaya reservoir insertion are often used in the management of acute hydrocephalus (9). The Ommaya reservoir is placed under the scalp and the other end is placed in the ventricle. The Ommaya reservoir allows for intermittent aspiration of CSF and drug delivery into the CSF to treat hydrocephalus, intracranial infection, or ventricular hemorrhage (10, 11). C-EVD improves the symptoms of hydrocephalus in emergency situations but cannot usually effectively control intracranial infection in a short term, while long-term C-EVD devices may cause retrograde intracranial infection, which may aggravate the condition (12, 13). To reduce the risk of postoperative infection, we adjusted the position of the outer end of the drainage tube in C-EVD to perform modified external ventricular drainage (M-EVD) and compared the surgical efficacy of M-VED with the Ommaya reservoir.

To our knowledge, the effects of M-EVD and an Ommaya reservoir in the management of hydrocephalus with intracranial infection (HII) in pediatric patients have not been compared. The purpose of the present study is to determine which surgical method has better therapeutic effects, is safer, and is more suitable for HII based on clinical, CSF, and outcome data.

## Materials and methods

This retrospective controlled study included 45 pediatric patients with HII treated with an Ommaya reservoir or M-EVD at Hebei Children's Hospital from January 2018 to December 2022. The institutional review board approved the study, and the parents of all pediatric patients provided informed consent for study inclusion.

This study has the following inclusion criteria: (1) patient age ranging from 1 to 3 years; (2) purulent meningitis and clinical manifestations: symptoms of intracranial hypertension (vomiting, headache, and papilla edema) or meningeal irritation (neck rigidity, positive Kernig's sign, and positive Brudzinski's sign); (3) hydrocephalus (bilateral lateral ventricular dilatation) confirmed on cephalic CT or MRI; (4) elevated CSF white blood cell count (WBC) and protein concentration (PR), and decreased CSF glucose concentration (GLU); (5) treatment with M-EVD or an Ommaya reservoir; and (6) parental provision of written informed consent for the operation and postoperative follow-up.

The exclusion criteria for this study are as follows: (1) patient age older than 3 years; (2) Hydrocephalus caused by ventricular hemorrhage; (3) open craniocerebral injury; (4) Iatrogenic intracranial infection; (5) genetic metabolic diseases; (6) intracranial space-occupying lesions; (7) treatment refusal or death; (8) Glasgow Coma Scale of 3 points (circulatory or respiratory circulatory failure); and (9) no informed consent provided by the legal representative of the pediatric patient.

A total of 52 patients with HII treated in the Department of Neurosurgery between January 2018 to December 2022 were screened for study eligibility. After 7 ineligible patients with HII were excluded, the total study cohort comprised 45 eligible patients with HII who received M-EVD or an Ommaya reservoir. There were 21 patients in the M-EVD group (14 male toddlers and 7 female toddlers; the median age 23.7 months) and 24 in the Ommaya reservoir group (15 male toddlers and 9 female toddlers; the median age 18.35 months). All included patients completed the designated follow-up.

### Clinical evaluation

The temperature and clinical symptoms of intracranial hypertension or meningeal irritation were recorded before and after the operation. Cephalic MRI or CT was performed to judge the degree of ventricular dilatation. The WBC, GLU, and PR in CSF were recorded pre- and postoperatively. Other recorded data were the time of resolution of the intracranial infection, time of VP shunt surgery, duration of hospitalization, complications, and outcomes.

The following criteria were met before extubation of the M-EVD and removal of the Ommaya reservoir to ensure that there were no other infectious foci: (1) the temperature was normal within 1–2 weeks; (2) the results of two consecutive CSF cultures were negative and the WBC was < 10^*^10^6^/L, indicating intracranial infection control; and (3) the patient had no symptoms of intracranial hypertension when the drainage tube was closed or no delayed CSF puncture in the Ommaya reservoir. When these criteria were met, the drainage tube or Ommaya reservoir was removed. VP shunt surgery was then performed if the patient had symptoms of intracranial hypertension.

### Operation procedures

#### Modified external ventricular drainage

After anesthesia, the patient is placed in a supine position with the head tilted to the left. Disinfect and lay sterile surgical sheet. The puncture point of the ventricle was 6 cm above the occipital tubercle and 3 cm to the right of the midline. An approximately 4-cm-long curved incision was made in the right occipital. A small hole (approximately 0.4-cm diameter) was drilled in the skull using a skull micro-dynamic system, and the dura mater was coagulated with bipolar electrocoagulation. An approximately 0.5-cm-long incision was made along the outer edge of the 10th rib. A subcutaneous tunnel was then created between the head and chest incisions using a rod. The shunt tube was led out through the tunnel. The ventricular end of the shunt tube was placed in the right ventricle perpendicular to an imaginary line connecting the bilateral external auditory canals. Then, 4 ml of CSF was extracted and sent to the laboratory for analysis. After the drainage tube was connected to the valve, the valve was pressed to check that there was CSF flowing out at the distal end of the drainage tube. The length of the external drainage tube at the chest wall was approximately 35 cm. The end of the drainage tube was connected to a closed drainage device. The surgical incision was sutured closed in layers.

#### Ommaya reservoir

The surgical procedure was similar to that used for M-EVD. The puncture point of the ventricle was 2.5 cm to the right of the midline and 1 cm in front of the coronal suture. The drainage tube was connected to the Ommaya reservoir and placed under the right side of the scalp. The CSF was suctioned smoothly by puncturing the Ommaya capsule with a No. 4.5 scalp needle.

### Postoperative care

Postoperatively, cephalic CT was performed to determine the position of the drainage tube in the ventricle and to check for bleeding in the ventricle. The drainage volume of CSF was generally limited to 100 ml. However, if the patient's condition was poor, the amount of CSF drainage was appropriately increased. Cefotaxime sulbactam sodium was used for empirical antibacterial therapy. After the etiological results were confirmed, sensitive antibiotics were used for intrathecal antibacterial therapy. After 8–10 days, the wound suture was removed. The CSF was re-examined every 3 days after surgery until the results of two consecutive CSF tests were normal. After the intracranial infection was controlled, the CSF drainage began to be restricted. The mental state of the patient was closely observed, and cephalic MRI or CT was intermittently repeated to judge the degree of hydrocephalus.

### Statistical analysis

SPSS 26.0 (IBM, Armonk, New York, USA) software was used to statistically analyze the preoperative and postoperative data of patients. Data collected during the last visit were used for comparisons with the preoperative baseline parameters, namely, the in-group comparisons. Additionally, between-group comparisons of two treatments at the last visit were performed. Continuous data were presented with mean ± standard deviation (X ± SD), and their normality status was detected by the Kolmogorov–Smirnov test, whereby the Student-*t* test was used for between-group comparisons and the paired Student-*t* test for in-group comparisons. Quartiles and non-parametric methods were used for calculating data that do not conform to a normal distribution. Categorical data were presented with numbers and percentages, and chi-square test or Fisher's exact test was used for between-group comparisons. A significance level of *P* < 0.05 was considered as statistically significant for all analyses.

## Results

There were no deaths in either of the two groups. There were no significant differences between the two groups in age and sex. No significant differences were found between the two groups in the admission temperature, weight, preoperative serum protein and albumin concentrations, CSF tests (WBC, GLU, and PR), and clinical symptoms (*P* > 0.05) (Table 1).

**Table 1:** Comparison of measurement data between M-EVD and Ommaya reservoir.

**Variable**		**M-EVD**	**Ommaya reservoir**	***P***
Age (Months)		23.7 (18.25, 34.85)	18.35 (15.78, 34)	0.285
Gender	Male	14 (66.67%)	15 (62.50%)	0.771
	Female	7 (33.33%)	9 (37.50%)	
Admission temperature		38.85 ± 0.46	38.85 ± 0.45	0.986
Weight (Kg)		11.8 (11.4, 14.8)	11.5 (10.65, 14.2)	0.275
Serum protein		42.63 ± 5.33	41.65 ± 6.35	0.554
Albumin		63.88 (54.82, 69.46)	62.48 (56.26, 72.09)	0.474
**Clinical symptoms**				
Intracranial hypertension	Positive	17 (80.95%)	19 (79.17%)	1.000
	Negative	4 (19.05%)	5 (20.83%)	
Meningeal irritation	Positive	15 (71.43%)	18 (75.00%)	0.787
	Negative	6 (28.57%)	6 (25.00%)	
Infection control time (Day)		19 (13, 23)	21 (14, 27.5)	0.226
V-P shunt (cases)	Operation	13 (61.90%)	15 (62.50%)	0.967
	Non	8 (38.10%)	9 (37.50%)	
Hospitalization time	< 30 days	9 (42.86%)	9 (37.50%)	0.723
	30–60 days	10 (47.62%)	14 (58.33%)	
	>60 days	2 (9.52%)	1 (4.17%)	

Albumin: 60–80 g/L. Serum Protein: 35–55 g/L. “Age, weight, albumin, infection control time, and hospitalization time” are quantitative variables that do not follow a normal distribution, and non-parametric tests are used. “Admission temperature and serum albumin” are quantitative variables that follow a normal distribution, and independent sample *t-*tests are used. The data of “gender, symptoms of intracranial hypertension, meningeal irritation and V-P shunt cases” were classified variables, and chi-square test was used.

### Cerebrospinal fluid

There were significant pre- vs. post-operative differences in the WBC, GLU, and PR of the CSF in both groups (*P* < 0.05) (Table 2). The median time taken for the CSF test results to return to normal was similar in the Ommaya reservoir group and the M-EVD group (19 vs. 21 *P* = 0.226) (Table 1).

**Table 2:** Comparison of CSF indexes.

	**M-EVD (*****n** **=*** **21)**			**Ommaya reservoir (*****n** **=*** **24)**			**Between-group comparison**
	**Preoperative**	**Postoperative**	*P* ^#^	**Preoperative**	**Postoperative**	*P* ^#^	*P* ^*^
WBC	198 (136, 284.5)	5 (3, 6.5)	0.000	162 (83.25, 248.25)	6 (5, 7)	0.000	0.311
GLU	1.24 (0.66, 2.01)	3.08 (2.71, 3.47)	0.000	1.29 (0.53, 1.79)	2.92 (2.04, 3.45)	0.000	0.524
PR	1.48 (1.18, 2.15)	0.39 (0.29, 0.49)	0.000	1.65 (1.24, 2.77)	0.32 (0.18, 0.41)	0.000	0.236

WBC: 0–10^*^10^6^/L, GLU: 2.5–4.4 mmol/L, PR: 0.2–0.4 g/L. ^#^Within-group comparison between preoperative and postoperative outcome measurement. ^*^Comparison of preoperative data between-group.

### Modified external ventricular drainage and the Ommaya reservoir

The drainage tube was closed or the puncture of the Ommaya reservoir was stopped after the attainment of two consecutive normal CSF test results.

In the Ommaya reservoir group, nine patients had no discomfort after the puncture was stopped, and the Ommaya reservoir was taken out a year later. In the Ommaya reservoir group, 15 patients developed symptoms of intracranial hypertension at 17.7 ± 6.5 days after the puncture was stopped, and these patients ultimately underwent VP shunt surgery.

In the M-EVD group, nine patients had no symptoms of intracranial hypertension after the drainage tube was closed and no ventricular dilatation was found on cephalic MRI. No patient reported discomfort after the M-EVD device was removed. However, one patient experienced a recurrence of hydrocephalus 8 months after the operation; the symptoms of hydrocephalus resolved after VP shunt surgery. After closing the drainage tube, symptoms of intracranial hypertension developed in 12 patients for an average duration of 14.9 ± 7.1 days, and the imaging results indicated ventricular dilatation. The symptoms resolved after VP shunt surgery. The final number of patients in the M-EVD group who underwent VP shunt surgery was 13.

### Complications

#### Ommaya reservoir group

At the scalp puncture site, two patients had redness and swelling that resolved after local disinfection. Additionally, one patient developed an unexplained blockage 19 days after the operation, and the CSF could not be successfully aspirated; when the intracranial infection was controlled, VP shunt surgery was performed.

One patient developed a scalp infection at the shunt valve about 2 years after VP shunt surgery. The infection did not improve with routine dressing changes. The scalp gradually developed defects, and the shunt valve was exposed. After full communication with the parents, the VP shunt was removed. The mental state of the patient was closely monitored during hospitalization. If the patient developed symptoms of intracranial hypertension and the results of the cephalic CT also indicated widened ventricles, then a second VP shunt would be performed. However, during the postoperative follow-up, the patient had no uncomfortable symptoms; consequently, the patient did not require a second operation.

#### M-EVD group

At 2 months after surgery, one patient still had not removed the drainage, as the CSF collected from the drainage tube showed abnormal results. All three CSF cultures grew *Streptococcus pneumoniae*. However, the patient had no signs of meningeal irritation and had a normal temperature. Therefore, we consider that *S. pneumoniae* had colonized the drainage tube. We performed a lumbar puncture to extract CSF, obtained normal CSF test results, and observed no bacterial growth in the CSF culture. Subsequently, the patient underwent VP shunt surgery and was in a very stable condition postoperatively.

Finally, three patients showed redness and exudation at the chest wall incision that improved after dressing changes and anti-infection treatment.

## Discussion

Central nervous system infections are mainly divided into hematogenous infections, in which pathogenic bacteria are transmitted through the bloodstream to invade brain tissue, direct infections, in which open intracranial injuries or infections such as sinusitis lead to intracranial infections, and iatrogenic infections, which are caused by neurosurgery-related procedures. Purulent meningitis can cause circulatory disturbance of CSF, disrupt the balance between the production and absorption of CSF, and thus lead to ventricular dilatation (8). For pediatric patients with HII, VP shunt surgery is only feasible when the intracranial infection is well controlled (14). Therefore, it is important for clinical doctors to know how to effectively control intracranial infection while alleviating hydrocephalus symptoms and reducing brain tissue damage (15). The present study is the first to compare the curative effects of M-EVD and Ommaya reservoir insertion for HII in pediatric patients. Both methods achieved good results. There were no significant differences between the two surgical methods in overall clinical efficacy and complications; however, each method has different advantages.

The conventional puncture point of the ventricle is 2.0 cm to the right of the midline and 2.5 cm posterior to the coronal suture. However, as this position is close to the ventricle, external bacteria may travel in a retrograde manner into the ventricle through the drainage tube, and long-term drainage may aggravate the intracranial infection. Therefore, a C-EVD tube is usually not retained for more than 7–10 days (16, 17). In contrast, the drainage tube in M-EVD is located at the lateral edge of the 10th rib, which is relatively far from the ventricle and thus greatly decreases the risk of retrograde intracranial infection. In both C-EVD and M-EVD, local infection at the outlet of the drainage tube may be cured by dressing changes. In the present study, one patient had the drainage tube retained for 61 days due to the presence of colonizing bacteria in the drainage tube. This patient had the longest drainage tube retention time in the present study. In hindsight, we should have performed a lumbar puncture and tested the CSF as soon as possible. However, from another perspective, this also demonstrates the feasibility and effectiveness of M-EVD because there was no retrograde intracranial infection during drainage for up to 2 months. As only one patient had a drainage tube in place for a long time, this does not prove that the drainage tube in M-EVD can be retained for several months. However, compared with C-EVD, M-EVD may allow the prolongation of the drainage time and seems to have better safety and effectiveness.

The Ommaya reservoir was originally used to deliver chemotherapy drugs to intracranial tumors. The purpose of the treatment is achieved by directly injecting drugs into the ventricle or tumor (18). With continuous advancements in disease diagnosis and treatment, the Ommaya reservoir is now used in the treatment of various nervous system diseases (19). The main advantage of the Ommaya reservoir is that it can be used for a long time, even for several years, and can be repeatedly punctured and drained (10, 11). The Ommaya reservoir is also an effective way to treat HII. Hydrocephalus cannot be effectively controlled in a short time. When the intracranial infection is controlled, CSF still needs to be intermittently extracted to relieve the symptoms of hydrocephalus. However, repeated puncture of the Ommaya reservoir increased the pain experienced by the pediatric patients in the present study. The scalp of a young child is thin, and repeated punctures may cause needle hole leakage and even skin ulceration. In the present study, two patients (8.3%) in the Ommaya reservoir group developed redness and swelling at the puncture site. Furthermore, one patient developed blockage of the Ommaya reservoir; when the Ommaya reservoir was removed in a second operation, the tube was blocked by some brain tissue, which made it impossible to extract CSF smoothly. Ihara (10) and Ye et al. (20) also reported local infection and CSF leakage after long-term puncture of an Ommaya reservoir. In contrast, to drain the CSF after M-EVD, the clinician only needs to turn the switch of the shunt tube. However, excessive CSF drainage should be avoided and the amount of extracted CSF should be measured daily. Furthermore, although M-EVD extends the drainage time, it cannot be used indefinitely. The drainage tube should be removed as soon as possible after intracranial infection control is achieved. While the Ommaya reservoir can remain in place for many years, it is recommended to remove the Ommaya reservoir as soon as possible once the symptoms of HII are improved (21). As with all implants, there are certain risks associated with the retention of the Ommaya reservoir for a long time. Overall, the Ommaya reservoir can be kept in place for longer than an external drainage tube (22, 23).

M-EVD and the Ommaya reservoir have the following advantages in treating HII: drainage or puncture can discharge a large number of pathogenic bacteria and inflammatory factors from the CSF, reduce the bacterial concentration in the CSF, accelerate CSF circulation, reduce intracranial pressure, and alleviate the symptoms of hydrocephalus. Both surgical methods also enable the intrathecal injection of antibiotics, thereby crossing the blood-brain barrier (24). After the drug enters the ventricle and subarachnoid space, it quickly spreads on the surface of the brain and can rapidly reach the desired treatment concentration in the infected area. This enables both treatment methods to effectively control infection, alleviate infection symptoms, strive for a short surgical treatment time, and improve the prognosis of the patient. Before the culture and sensitivity results were confirmed, all patients in the present study were treated with empirical anti-infection therapy with cefotaxime sulbactam sodium. Intrathecal antibacterial medication (vancomycin) was used for anti-infection treatment after the operation. Once the culture and sensitivity results were known, the most sensitive antibiotic was administered intrathecally. After intrathecal injection, the drainage tube of M-EVD should be closed or the Ommaya reservoir puncture should be suspended for 2 h to ensure that the drugs are evenly distributed on the brain surface through CSF circulation (25–27). Related studies report that different drugs injected intrathecally may cause different adverse reactions. For example, vancomycin may cause temporary hearing loss, polymyxin may cause meningitis reactions and epilepsy, and a high concentration of meropenem may induce seizures (28, 29). In the present study, nine patients experienced headache during intrathecal injection of medication, but the headache symptoms disappeared after the injection was completed. As the infection was gradually controlled, the headache symptoms during the injection process gradually decreased.

There is no open intracranial injury and no surgical history in the case data included in this study. The common bacteria in cerebrospinal fluid culture are *Streptococcus pneumoniae, Staphylococcus aureus, Klebsiella pneumoniae, Haemophilus influenzae*, and *Escherichia coli*. A small proportion of the results were *Listeria monocytogenes* and *Acinetobacter Qilenbao*. Most cerebrospinal fluid cultures showed no bacterial growth. In addition, some parents could not remember whether there had been recent upper respiratory infections or digestive system diseases. However, it is more likely to consider blood-borne infection.

This study was subject to several limitations. First, the retrospective design might have impeded the accuracy and precision in data collection. Second, due to the limited use in our institution, only 45 eligible patients were included for data analysis, making the comparison not definitely conclusive. It was possible the true differences between two treatments for some outcome variables were hampered by limited statistical power caused by small sample size, which was known as type II statistical error. Third, the single-center design would have lowered the generalizability of our results to other settings. Fourth, if there are multiple abnormalities in the cerebrospinal fluid results in the drainage tube, lumbar puncture should be performed to obtain CSF in time and the results should be compared.

## Conclusion

Both M-EVD and Ommaya reservoir implantation effectively reduce the intracranial pressure and alleviate the symptoms of hydrocephalus. Intrathecal antibacterial medications can quickly reach appropriate drug concentrations and treat intracranial infections. The main advantage of M-EVD over C-EVD is that it prolongs the retention time of the drainage tube and reduces the risks of drainage tube displacement and detachment, CSF leakage, and retrograde intracranial infection. The disadvantage of Ommaya reservoir implantation is that repeated scalp punctures may cause a local inflammatory reaction; however, the device can stay in place for a longer period than the drainage tube in M-EVD.

## Data availability statement

The raw data supporting the conclusions of this article will be made available by the authors, without undue reservation.

## Ethics statement

The studies involving human participants were reviewed and approved by the Institutional Review Board of Hebei Children's Hospital. Written informed consent to participate in this study was provided by the patients/participants or patient/participants' legal guardian/next of kin. Written informed consent was obtained from the individual(s) and/or minor(s)' legal guardian/next of kin for the publication of any potentially identifiable images or data included in this article.

## Author contributions

LC: Conceptualization, Writing – original draft. MH: Data curation, Writing – original draft. LS: Data curation, Methodology, Writing – original draft. YY: Investigation, Software, Writing – review & editing. PL: Investigation, Software, Writing – review & editing. JF: Investigation, Software, Writing – original draft. NW: Resources, Writing – review & editing. ZC: Resources, Supervision, Writing – review & editing. YQ: Methodology, Software, Writing – original draft. ZY: Resources, Supervision, Writing – review & editing. YS: Writing – original draft, Writing – review & editing.
